# Quantification and Assessment of Interfraction Setup Errors Based on Cone Beam CT and Determination of Safety Margins for Radiotherapy

**DOI:** 10.1371/journal.pone.0150326

**Published:** 2016-03-01

**Authors:** Macarena Cubillos Mesías, Judit Boda-Heggemann, Johannes Thoelking, Frank Lohr, Frederik Wenz, Hansjoerg Wertz

**Affiliations:** Department of Radiation Oncology, Universitätsmedizin Mannheim, Medical Faculty Mannheim, Heidelberg University, Mannheim, Germany; The University of Chicago, UNITED STATES

## Abstract

**Introduction:**

To quantify interfraction patient setup-errors for radiotherapy based on cone-beam computed tomography and suggest safety margins accordingly.

**Material and Methods:**

Positioning vectors of pre-treatment cone-beam computed tomography for different treatment sites were collected (n = 9504). For each patient group the total average and standard deviation were calculated and the overall mean, systematic and random errors as well as safety margins were determined.

**Results:**

The systematic (and random errors) in the superior-inferior, left-right and anterior-posterior directions were: for prostate, 2.5(3.0), 2.6(3.9) and 2.9(3.9)mm; for prostate bed, 1.7(2.0), 2.2(3.6) and 2.6(3.1)mm; for cervix, 2.8(3.4), 2.3(4.6) and 3.2(3.9)mm; for rectum, 1.6(3.1), 2.1(2.9) and 2.5(3.8)mm; for anal, 1.7(3.7), 2.1(5.1) and 2.5(4.8)mm; for head and neck, 1.9(2.3), 1.4(2.0) and 1.7(2.2)mm; for brain, 1.0(1.5), 1.1(1.4) and 1.0(1.1)mm; and for mediastinum, 3.3(4.6), 2.6(3.7) and 3.5(4.0)mm. The CTV-to-PTV margins had the smallest value for brain (3.6, 3.7 and 3.3mm) and the largest for mediastinum (11.5, 9.1 and 11.6mm). For pelvic treatments the means (and standard deviations) were 7.3 (1.6), 8.5 (0.8) and 9.6 (0.8)mm.

**Conclusions:**

Systematic and random setup-errors were smaller than 5mm. The largest errors were found for organs with higher motion probability. The suggested safety margins were comparable to published values in previous but often smaller studies.

## Introduction

Radiation therapy has experienced an evolution from the 2-D approach to 3-D techniques, from conformal to intensity-modulated radiotherapy (IMRT), increasing the accuracy of dose delivery on the target volume and sparing the normal tissues. Due to this high conformity and rapid dose fallout outside the tumor a high geometric accuracy of the daily dose delivery is essential [1]. The introduction of image-guided radiation therapy (IGRT) allows a very accurate determination of the position of the clinical target volume (CTV) and organs at risk during the course of the treatment [2] and therefore has the potential to decrease the size of the planning target volume (PTV) to CTV margin in order to reduce the dose to normal tissue. There are multiple available image-guidance systems using ionization radiation or other technologies [3]. One frequently used system is kilovoltage cone-beam computer tomography (kV CBCT), which provides a 3-D visualization of the structures with soft tissue contrast [4, 5].

One important source of geometrical uncertainty in the radiotherapy process are interfractional setup errors during patient positioning which can be defined as a discrepancy between the anatomy of the patient at the planning CT and at the treatment. These errors can be divided into systematic errors (which are reproducible consistent errors, occurring in the same direction and magnitude) and random errors (which vary in direction and magnitude). Whereas the random errors blur the dose distribution, the systematic errors cause a shift of the cumulative dose distribution [6]. Because the interfractional setup errors play an important role for the overall treatment success, one major requirement during radiotherapy is to reduce these errors as far as possible and to introduce safety margins around the CTV to compensate for the remaining geometric uncertainties. However this safety margin, i.e. the CTV to PTV expansion is by definition occupied only by normal tissue. The aim of this work was to quantify the interfractional setup errors for different treatment sites guided by kV CBCT and suggest safety margins accordingly.

## Methods and Materials

This study was approved by the local ethics committee / Institutional Review Board (IRB) (Medizinische Fakultät Mannheim, Medizinische Ethik-Kommission II) (2015-859R-MA). Written informed consent was given by the participants for retrospective anonymized analyses. In addition, the patient information was anonymized and de-identified before analysis.

### Patient population and positioning based on kV CBCT

The data from 443 unselected patients treated in our institution between January 2013 and March 2014 with the following treatment sites were analyzed: prostate, prostate bed, cervix, rectum, anal, head and neck, brain and mediastinum. Prior to the treatment session a kV CBCT was performed for all patients at the treatment machine (Elekta, Sweden) in treatment position. The localization CBCT was matched with the original planning CT using bone matching and/or soft tissue-grey value matching followed by a manual correction if appropriate. The positioning correction vectors were calculated after the whole matching procedure including automated and manual user matching. and a translational isocenter correction by shifting the treatment couch was carried out.

### Data analysis

The positioning translational vectors of each treatment session were collected from the record and verify system (Mosaiq 2.5, Elekta, Sweden) resulting in a total of 9504 kV CBCTs. The data for each treatment site is shown in detail in Table 1. For each patient data set the individual average and standard deviation were calculated. Besides the total average the standard deviation, minimum and maximum value for each treatment site were computed. Additionally, the overall mean error (M), systematic error (Σ) and random error (σ) were determined according to van Herk’s formalism: M is defined as the mean of all individual means, Σ corresponds to the standard deviation of all individual means, and σ is calculated through the root mean square of the individual standard deviations of all patients [6]. For prostate, prostate bed, head and neck, brain and mediastinum, in which the kV CBCTs were realized on a daily basis, the calculations were carried out also for the first 5 sessions. For cervix, rectum and anal the CBCT in our institution were done only once a week. Therefore, this first 5 fraction analysis was not performed for these treatment sites. Finally, a CTV-to-PTV margin expansion (if no image guidance system was used) was computed using van Herk’s equation (2.5Σ + 0.7σ).

**Table 1:** Number of patients and CBCTs per treatment site.

Treatment site	Number of patients	Number of CBCT
Prostate	63	1615
Prostate Bed	51	1339
Cervix	16	306
Rectum	43	339
Anal	30	235
Head and Neck	99	2851
Brain	88	1583
Mediastinum	53	1236
Total	443	9504

## Results

**Fig 1:**
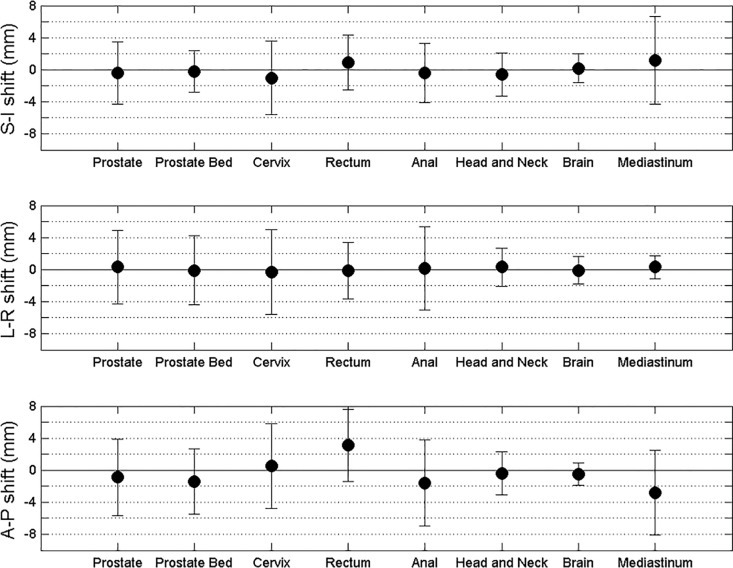
Average shifts and standard deviations for treatment sites in superior-inferior (SI), left-right (L-R) and anterior-posterior (A-P) direction.

The average shifts and standard deviations for all the treatment sites in all three directions are shown in Fig 1.

The results of M, Σ and σ in all three directions are presented in Table 2 followed by a comparison with the results found in the literature [7–21]. For the systematic error, the largest value could be found in mediastinum on the three directions, whereas the smallest values were found in brain. For random error, the largest value was found for anal in the L-R direction (5.1mm), whereas the smallest values were also found in brain. In overall, the values were smaller than 5mm with a mean (SD) of 2.1 (0.7), 2.1 (0.5) and 2.5 (0.8)mm for the systematic error and 2.9 (0.9), 3.3 (1.2) and 3.2 (1.2)mm for the random error in superior–inferior (S-I), left-right (L-R) and anterior-posterior (A-P) direction, respectively.

**Table 2:** Overall mean (M), systematic (Σ) and random setup errors (σ) / comparison with published data.

Treatment Site	n	CBCTs	Modality	M (mm)			Σ (mm)			σ (mm)		
				S-I	L-R	A-P	S-I	L-R	A-P	S-I	L-R	A-P
**Prostate**												
Present study	63	1615	kV CBCT	-0.6	0.3	-1.1	2.5	2.6	2.9	3	3.9	3.9
Wong *et al*.	329	1870	kV CT	n.d.	n.d.	n.d.	1.7	2.3	4.3	2.3	3.2	3.9
Snir *et al*.	17	449	kV CBCT	0.4	-0.9	0.6	1.8	1.6	2.6	1.8	2.3	3
Mayyas *et al*.	27	1100	kV CBCT	0.2	1.1	-1.2	2.7	2.4	3	2.2	2.5	3.2
Qi *et al*.	36	957	kV CBCT	n.d.	n.d.	n.d.	1.8	1.8	2.1	2.3	2.1	2.8
	36	1097	MV CT	n.d.	n.d.	n.d.	1.7	1.9	2.1	1.8	3.5	3.5
**Prostate Bed**												
Present study	51	1339	kV CBCT	-0.4	-0.1	-1.5	1.7	2.2	2.6	2	3.6	3.1
Ost *et al*.	15	547	kV CBCT	-0.5	1.5	1.7	2	2.7	2.7	1.5	2	2.3
Huang *et al*.	14	420	kV CBCT	-0.9	0	1.9	1.3	1	2.5	3.1	1	3.1
**Cervix**												
Present study	16	306	kV CBCT	-1	-0.3	0.4	2.8	2.3	3.2	3.4	4.6	3.9
Santanam *et al*.	10	310	MV CT	-3	-1.3	1.3	4.6	2	1.5	4.8	3.4	3.7
**Rectum**												
Present study	43	339	kV CBCT	0.8	0.1	2.8	1.6	2.1	2.5	3.1	2.9	3.8
**Anal**												
Present study	30	235	kV CBCT	-0.2	0.4	-1.8	1.7	2.1	2.5	3.7	5.1	4.8
Chen *et al*.	20	365	MV CBCT	-2.3	2.1	1.1	3.2	3.6	1.1	2.9	5.5	3.8
**Head and Neck**												
Present study	99	2851	kV CBCT	n.d.	n.d.	n.d.	1.9	1.4	1.7	2.3	2	2.2
Den *et al*.	28	1013	kV CBCT	n.d.	n.d.	n.d.	1.1	1.1	1.4	2	1.5	1.9
Velec *et al*.	11	338	kV CBCT	n.d.	n.d.	n.d.	1	0.8	1	1.5	1.5	1.6
Qi *et al*.	29	632	kV CBCT	n.d.	n.d.	n.d.	1.8	1	1.2	1.6	1.4	1.4
	35	974	MV CT	n.d.	n.d.	n.d.	2.3	1.2	1.1	1.7	1.7	1.7
	53	1696	MV CBCT	n.d.	n.d.	n.d.	1.8	1.9	3	2.1	2	2
**Brain**												
Present study	88	1583	kV CBCT	0.2	-0.1	-0.5	1	1.1	1	1.5	1.4	1.1
Tryggestad *et al*.	20	462	kV CBCT	-0.5	0.6	-0.3	1.2	1	1.1	1.4	1.2	1.1
**Mediastinum**												
Present study	53	1236	kV CBCT	1	0.6	-2.5	3.3	2.6	3.5	4.1	3.1	2.6
Borst *et al*.	62	524	kV CBCT	-1.3	0	0.3	1.9	1.7	1.2	3.8	3.1	1.4
Grills *et al*.	24	308	kV CBCT	n.d.	n.d.	n.d.	2.9	2	5.8	3.5	2.7	2
Yeung *et al*.	13	389	kV CBCT	3.7	2.4	-2.4	5.6	3.5	3.2	4.6	3.7	4

Abbreviations: S-I = superior-inferior; L-R = left-right; A-P = anterior-posterior; n.d. = not determined.

A comparison of the overall treatment and the first 5 sessions is shown in Table 3. It can be seen that the differences are smaller than 1mm. The largest differences were found in the systematic error in prostate (S-I direction) and prostate bed (L-R direction) with a value of 0.9mm. For the random error the largest value was found also in S-I direction of prostate (0.5mm).

**Table 3:** Systematic and random setup errors for the total and first 5 sessions.

Treatment site	Direction	Σ (mm)			σ (mm)		
		Total	5 sessions	|Diff|	Total	5 sessions	|Diff|
Prostate	S-I	2.5	3.4	0.9	3	2.5	0.5
	L-R	2.6	3.3	0.7	3.9	3.8	0.1
	A-P	2.9	3.7	0.8	3.9	3.6	0.3
Prostate Bed	S-I	1.7	1.7	0	2	2.1	0.1
	L-R	2.2	3.1	0.9	3.6	3.3	0.3
	A-P	2.6	2.9	0.3	3.1	2.8	0.3
Head and Neck	S-I	1.9	2.6	0.7	2.3	2.1	0.2
	L-R	1.4	1.7	0.3	2	1.8	0.2
	A-P	1.7	2	0.3	2.2	2	0.2
Brain	S-I	1	1.2	0.2	1.5	1.4	0.1
	L-R	1.1	1.3	0.2	1.4	1.4	0
	A-P	1	1	0	1.1	1.1	0
Mediastinum	S-I	3.3	3.7	0.4	4.6	4.4	0.2
	L-R	2.6	3.2	0.6	3.7	3.7	0
	A-P	3.5	3.4	0.1	4	3.6	0.4

Abbreviations: S-I = superior-inferior; L-R = left-right; A-P = anterior-posterior; Σ = systematic error; σ = random error; |Diff| = absolute value of the difference.

For each treatment site the calculated CTV-to-PTV expansion values for the overall sessions are presented in Table 4. The largest values could be found for mediastinum and the smallest for brain. These results were expected because of the values of systematic and random errors computed previously.

**Table 4:** Calculated clinical target volume (CTV) to planning target volume (PTV) margin expansion.

Treatment site	Direction (mm)		
	S-I	L-R	A-P
Prostate	8.4	9.2	10
Prostate Bed	5.7	8	8.7
Cervix	9.4	9	10.7
Rectum	6.2	7.3	8.9
Anal	6.8	8.8	9.6
Head and Neck	6.4	4.9	5.8
Brain	3.6	3.7	3.3
Mediastinum	11.5	9.1	11.6

Abbreviations: S-I = superior.inferior; L-R = left-right; A-P = anterior-posterior.

## Discussion

Interfraction setup errors for 8 different treatment sites were analyzed retrospectively using 9504 CBCT studies and a CTV-to-PTV margin expansion was proposed. The results showed that the variation was large for pelvis and thorax and small for head and neck treatments. This can be explained due to the use of immobilization devices for both head and neck and brain treatments and the presence of the skull in brain tumors which restricts the daily motion of the structures.

When analyzing the values for mean, systematic and random error, a larger value for M was found for rectum and mediastinum in the A-P direction. The mean value could deviate from zero due to some imprecisions in the setup procedure [6]. In our institution the rectum treatments are performed with the patient in prone position using a belly board whereas mediastinum in supine position with the arms up and a wing board. However, the values we obtained for mean, systematic and random error are similar to the values found in the literature.

For the cases where the values for the overall and the first 5 sessions were calculated, it can be seen that the differences are within the tolerance levels for geometrical alignment, i.e., less than 1mm [22], with the smallest differences were found in brain and the largest in prostate. Therefore, it is plausible to assume that the image guidance could be performed the first 5 sessions and the rest of the treatment with the skins or mask marks for selected cases. However, due to the highly precise radiation delivery required for IMRT treatments, there is a potential risk of overdose in the organs at risk, especially those adjacent to the PTV. Therefore, it is a clinical decision to assume the risk.

Because we analyzed a huge amount of data over a longer time period for different linacs we can not exclude that there was a recalibration of the CBCT performed between different datasets. However, whenever a CBCT recalibration is performed it is guaranteed that the original accuracy according to the acceptance procedure of the linac and the CBCT system is restored. The recalibration is performed with a standard ball bearing phantom. During the calibration procedure the small spherical phantom is even positioned with an accuracy of few tenths of a millimetre (mostly ~0.2mm) in the MV isocenter before the new flexmaps of the CBCT and the kV isocenter are defined. Therefore the magnitude of a deviation before and after the recalibration (introduction of a new systematic error) of the CBCT system is in submillimeter range and was not separately taken into account.

One limitation of this study is that it does not account for residual errors because a second (verification) CBCT scan after the repositioning was not carried out. In other studies, for prostate bed [11] and head and neck [23] the residual errors were determined by performing a second CBCT after the treatment and matching it with the planning CT. Of course this procedure will also lead to a higher dose exposure to the patient. The assessment of the residual errors will allow to calculate CTV-to-PTV expansion margins when image guidance is used which should in general be smaller than the values obtained in this study. Because safety margins are usually applied in three dimensions even a small reduction can result in a considerably reduced normal tissue volume.

## Conclusions

The systematic and random errors found in this work using IGRT in a clinical routine setup were not larger than 5mm for the different treatment sites. When the overall treatment is compared with the first 5 sessions the results for the interfraction positioning errors do not show a large variability. Besides, the CTV-to-PTV margin expansions were calculated based on a large number of CBCT based repositioning vectors. The margins were not larger than 12mm, similar to literature values and thus confirming previous studies. A future work could be initiated to assess the residual errors after position correction to suggest reduced margins when IGRT is performed.
